# A novel soybean hairy root system for gene functional validation

**DOI:** 10.1371/journal.pone.0285504

**Published:** 2023-05-18

**Authors:** Bruna Medeiros Pereira, Fabrício Arraes, Andressa Cunha Quintana Martins, Nayara Sabrina Freitas Alves, Bruno Paes Melo, Carolina Vianna Morgante, Mario Alfredo Passos Saraiva, Maria Fátima Grossi-de-Sá, Patricia Messenberg Guimaraes, Ana Cristina Miranda Brasileiro

**Affiliations:** 1 EMBRAPA Recursos Genéticos e Biotecnologia, Brasília, DF, Brazil; 2 Instituto Nacional de Ciência e Tecnologia—INCT PlantStress Biotech-Embrapa, Brasília, DF, Brazil; 3 EMBRAPA Semiárido, Petrolina, PE, Brazil; National Taiwan University, TAIWAN

## Abstract

*Agrobacterium rhizogenes*-mediated transformation has long been explored as a versatile and reliable method for gene function validation in many plant species, including soybean (*Glycine max*). Likewise, detached-leaf assays have been widely used for rapid and mass screening of soybean genotypes for disease resistance. The present study combines these two methods to establish an efficient and practical system to generate transgenic soybean hairy roots from detached leaves and their subsequent culture under *ex vitro* conditions. We demonstrated that hairy roots derived from leaves of two (tropical and temperate) soybean cultivars could be successfully infected by economically important species of root-knot nematodes (*Meloidogyne incognita* and *M*. *javanica*). The established detached-leaf method was further explored for functional validation of two candidate genes encoding for cell wall modifying proteins (CWMPs) to promote resistance against *M*. *incognita* through distinct biotechnological strategies: the overexpression of a wild *Arachis* α-expansin transgene (*AdEXPA24*) and the dsRNA-mediated silencing of an endogenous soybean polygalacturonase gene (*GmPG*). *AdEXPA24* overexpression in hairy roots of RKN-susceptible soybean cultivar significantly reduced nematode infection by approximately 47%, whereas *GmPG* downregulation caused an average decrease of 37%. This novel system of hairy root induction from detached leaves showed to be an efficient, practical, fast, and low-cost method suitable for high throughput *in root* analysis of candidate genes in soybean.

## 1. Introduction

The soil Gram-negative *Agrobacterium rhizogenes* (revised as *Rhizobium rhizogenes* by [1] is the causative agent of hairy root disease that affects many higher plants, in particular dicotyledonous species [2, 3]. During the infection process, a DNA fragment (T-DNA) present in the large *A*. *rhizogenes* Ri plasmids is transferred and stably integrated into the host genome [4]. The subsequent expression of T-DNA genes in plant cells results in rhizogenic reactions at the site of bacterial infection, characterized by neoplastic and abnormal root growth, abundant proliferation of adventitious roots, profusion of root hairs, and an agravitropic phenotype (so-called ‘hairy roots’). With advances in understanding its molecular infection mechanism, the natural and unique ability of *A*. *rhizogenes* to cross-kingdom gene transfer has been extensively explored for plant genetic engineering for diverse biotechnological purposes [5, 6]. Furthermore, axenic cultures of hairy roots displayed several desirable features making them suitable approach for boosting the production of recombinant proteins, such as easy and efficient production from different explant tissues, fast, prolific and unlimited growth, simple culture of individual transgenic lines, rapid scaling up in bioreactors, and maintenance over a prolonged period. Therefore, hairy roots transformed by pathogenic *A*. *rhizogenes* strains have been widely used for enhanced production of secondary metabolites in medicinal plants, investigation of gene function, and general *in root* biology studies [7–10].

Soybean [*Glycine max* (L.) Merrill] is one of the most valuable crops worldwide, being Brazil the leading global producer, with an expected production for the 2020/2021 crop year of nearly 134 million metric tons (https://www.statista.com). The successful performance of soybean in Brazil is mainly due to the development in the 1980s of high-yielding cultivars, well adapted to the tropical region’s shorter day length and high aluminum and low calcium soils [11]. However, despite the boom in tropical soybean production, adverse environmental conditions, particularly drought periods and disease incidences, affect plant performance and are still yield-limiting factors with substantial economic impacts [12, 13]. Root-knot nematodes (RKN) *Meloidogyne* spp. are among the most important soybean pests and are particularly devastating in tropical and sub-tropical regions where they can cause severe yield losses [14]. In infested soybean planting areas, RKN are currently controlled by planting early-maturing cultivars, crop rotation, and application of nematicides that increase the cost of production and negatively affect the environment [15]. With these limited management options, the use of resistant cultivars is the most economical and environmentally friendly strategy for RKN control. However, genetic resistance is usually described as a complex trait with major gene resistance that is not often durable and limited natural sources of resistance [16]. In this context, there is an essential need to identify additional RKN-resistance sources aiming at the development of new high-yielding resistant soybean cultivars.

Cultivated and wild *Glycine* species are strongly susceptible to infection by *A*. *rhizogenes* [2], and hairy root transformation approaches have been widely explored for studies of soybean root-specific processes and interactions [17, 18]. To date, several genes putatively involved in nematode resistance have been characterized in soybean hairy roots through transgene overexpression or RNAi-mediated silencing strategies [19–24]. Most of these studies use composite plants as a model system consisting of wild-type non-transgenic shoots with transgenic hairy roots induced after inoculation with a wild *A*. *rhizogenes* strain harboring recombinant binary vectors. However, the generation of composite soybean plants is still a laborious, lengthy, and space-consuming process that involves handling whole fast-growing plants that take at least 16 days to generate hairy roots [17, 25]. Moreover, the maintenance of soybean composite plants is not feasible for extended periods. Over the last few years, we successfully developed and applied a large-scale method for *in root* functional characterization of candidate genes in nematode-susceptible peanut (*Arachis hypogaea*) [20, 26–28]. This *ex vitro* methodology has proven to be a simple, quick, cost-efficient, and space-saving solution for *in root* analysis, being a valuable tool to study peanut-RKN interaction without the need for nematode sterilization or maintenance of *in vitro* axenic hairy root cultures.

In the present study, we developed and optimized a new protocol to generate and cultivate transgenic soybean *ex vitro* hairy roots using detached leaves as explants by drawing on our previous research on peanut hairy roots. We also showed that two of the most ubiquitous and economically important RKN species (*M*. *incognita and M*. *javanica*) are capable of infecting and successfully completing their entire life cycle inside hairy roots derived from detached leaves from tropical and temperate soybean cultivars. As a proof of principle, the efficacy of this method for candidate gene functional validation was demonstrated by modulating the expression of genes encoding cell wall modifying proteins (CWMPs) in soybean hairy roots derived from detached leaves. Therefore, a gene encoding for an α-Expansin isolated from wild *Arachis duranensis* (*AdEXPA24*) was overexpressed, while an endogenous soybean polygalacturonase gene (*GmPG*) was silenced in transgenic soybean hairy roots. *AdEXPA24* belongs to the plant expansin superfamily involved in cell wall relaxation and remodeling and has been commonly associated with abiotic and biotic stress responses [30]. Besides, plant-endogenous Polygalacturonases are responsible for extensive pectin disassembly necessary for almost developmental activities, and its suppression could be associated with reduced pathogen susceptibility [31, 32]. Our results showed that both *AdEXPA24* overexpression and *GmPG* silencing resulted in a significant reduction in the number of galls in soybean hairy roots corroborating the efficiency of the detached leaves approach. By using distinct biotechnological strategies, this study reveals two CWMP-encoding genes as valuable candidates for genetic engineering toward enhanced resistance to RKN infection in soybean.

## 2. Materials and methods

### 2.1. Cloning of *AdEXPA24* gene for overexpression in hairy roots

The *Arachis duranensis EXPA24* gene (*AdEXPA24*) encoding for an α-Expansin was identified by our previous genome-wide analysis of *Arachis* expansin genes [21]. The complete coding sequence of *AdEXPA24* was determined by the alignment of Aradu.G235T gene model sequence (https://peanutbase.org/) with *Arachis* transcript sequences available at NCBI (http://www.ncbi.nlm.nih.gov/) and our previous *Arachis* transcriptome surveys [29, 33] using the default parameters of SnapGene® software (available at www.snapgene.com).

To overexpress *AdEXPA24* in soybean hairy roots, the deduced 759 bp consensus coding sequence was synthesized and cloned by Epoch Life Science Inc. (Missouri City, Texas, USA) into the unique *XhoI* site of the binary vector pPZP-BAR, under the control of the *Arabidopsis thaliana* actin 2 (AtACT2) promoter and the *Agrobacterium* nopaline synthase (NOS) terminator. The binary vector pPZP-BAR (hereafter called pPZP-empty; [29] is derived from pPZP_201BK_EGFP [34] and contains two cassettes for constitutive expression of *enhanced green fluorescent protein* (*eGFP*) reporter gene and the *bar* gene for glufosinate ammonium herbicide resistance. The vector generated was named pPZP-AdEXPA24.

### 2.2. Cloning of *GmPG* gene (dsRNA) for RNA interference in hairy roots

Our previous studies showed that orthologs of the *G*. *max* Polygalacturonase-encoding gene (*GmPG*; GenBank accession n°. Glyma.15G136700) are downregulated in response to *Meloidogyne* spp. infection in three RKN-resistant legume species [35]. Here, an RNAi-inducing binary vector was constructed using previously described parameters [36] to downregulate the expression of *GmPG* in soybean hairy roots. Briefly, different fragments (cutoff of 400 bp) of the *GmPG* coding sequence were inspected for their specificity using nucleotide BLAST (blast.ncbi.nlm.nih.gov/), and those with no hits against public databanks selected for cloning. The stability of the selected dsRNA molecules was checked through Mfold RNA secondary structure prediction software [37], and sequences showing the most negative difference in Gibb’s free energy were chosen. The homology of the selected dsRNA with non-target organisms was predicted by siRNA Target Finder (https://www.genscript.com/tools/sirna-target-finder) and dsCheck (http://dscheck.rnai.jp/) tools. The specific 400 bp sequence was then synthesized in sense and antisense orientations, separated by a PDK intron from the pRNAi-psiuK vector [36], and under the control of the constitutive promoter of CaMV35S and the NOS terminator, using the services of Epoch Life Science Inc. (Missouri City, Texas, USA). The entire cassette was then cloned into the binary vector pPZP-empty (hereafter called pPZP-siGmPG), as described above.

### 2.3. Preparation of *Agrobacterium rhizogenes* inoculum

The pPZP-empty, pPZP-AdEXPA24 and pPZP-siGmPG binary vectors were introduced into the cucumopine-type *A*. *rhizogenes* ‘K599’ wild strain using the standard electroporation protocol. Transformed colonies were selected by PCR using specific primer pairs from AdEXPA24, siGmPG and eGFP sequences (Table 1).

**Table 1:** Primers used for PCR and qRT-PCR analysis.

Primer name	Putative function	Forward primer (5’-3’)	Reverse primer (5’-3’)	Amplicon (bp)	Reference
**AdEXPA24**	α-Expansin	GAACATCACCAGCACCTCCA	ACCCTCCTTTGCAGCACTTT	192	[38]
**eGFP**	Green Fluorescent Protein	CGACCACATGAAGCAGCACGAC	TCCTCGATGTTGTGGCGGATCT	294	This study
**siGmPG**	Polygalacturonase	GAAAGAAGCTTGAATTCGGTACCC	GAAAGAAGCTTTTCGAACCCAGC	830	This study
**qsiGmPG**	Polygalacturonase	TCGATGTCAGGTCCTTTGGTG	TTTCCATGCTGCCACGAATG	74	This study
**GmCYP2**	Cyclophilin 2	CGGGACCAGTGTGCTTCTTCA	CCCCTCCACTACAAAGGCTCG	154	[39]
**GmELF1A**	Translation elongation factor 1α	GACCTTCTTCGTTTCTCGCA	CGAACCTCTCAATCACACGC	195	[39]
**SCAR-inc-K14**	SCAR marker for *Meloidogyne incognita*	GGGATGTGTAAATGCTCCTG	CCCGCTACACCCTCAACTTC	399	[40]
**SCAR-jav**	SCAR marker for *Meloidogyne javanica*	GGTGCGCGATTGAACTGAGC	CAGGCCCTTCAGTGGAACTATAC	670	[41]

**Fig 1:**
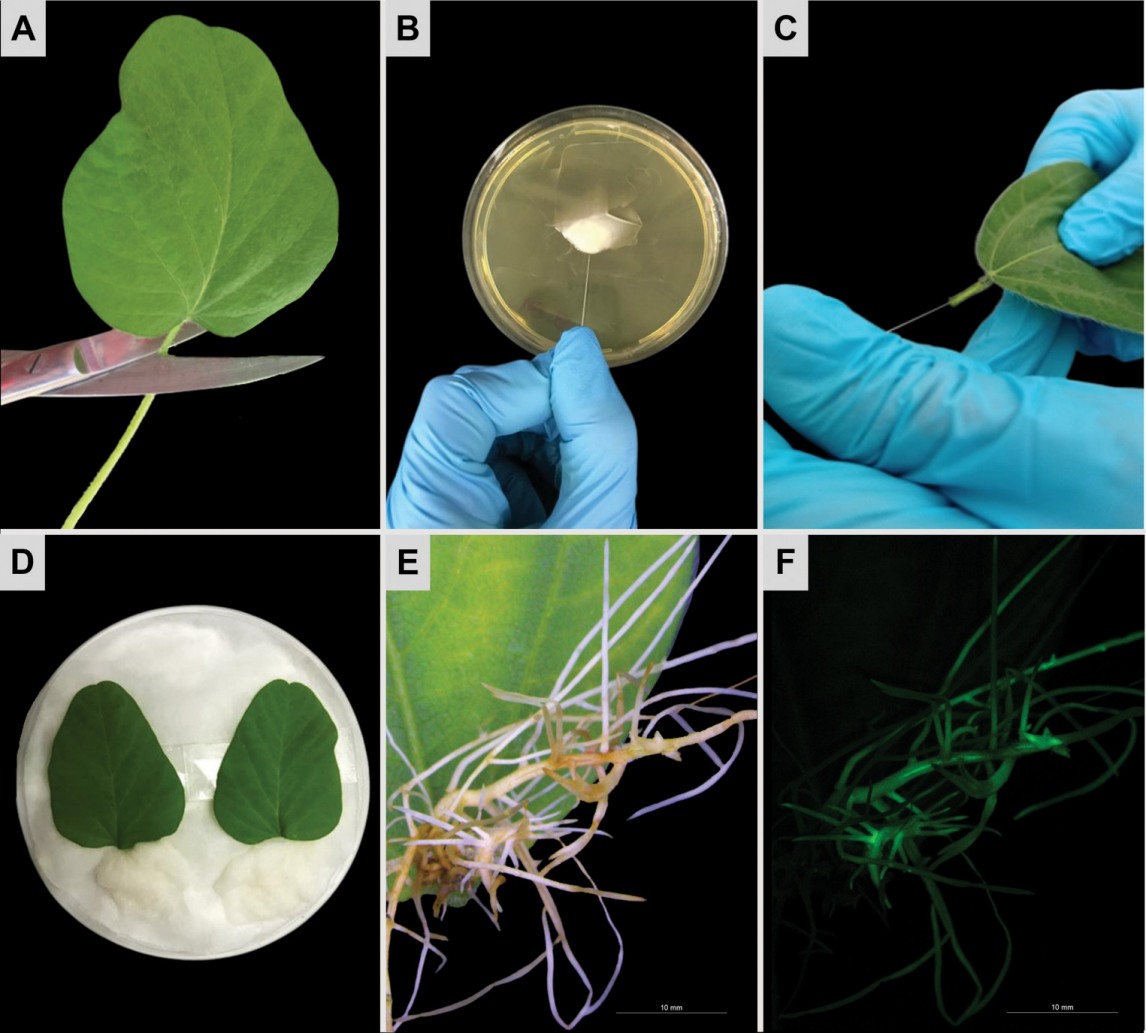
Hairy roots induced by *Agrobacterium rhizogenes*-mediated transformation in the detached leaves of soybean). **(A)** Young unifoliate leaf detached from a 20-day-old soybean (*Glycine max* cultivar ‘BRS 537’) plant grown under greenhouse conditions. **(B)** Picking on a fresh *A*. *rhizogenes* bacterial paste with a needle for **(C)** inoculation in the petiole of a unifoliate leaf. **(D)** Leaves placed in a Petri dish with the inoculated petiole covered by sterilized wet cotton. **(E, F)** Hairy roots emerged from soybean detached leaf imaged using **(E)** brightfield and **(F)** fluorescence stereomicroscopy, 20 days after *A*. *rhizogenes* transformation.

The *A*. *rhizogenes* ‘K599’ strain harboring the binary vector was inoculated directly from glycerol stock at -80°C into 1.6% (w/v) Luria-Bertani (LB) semi-solid culture medium with streptomycin (300 mg/L) and kanamycin (100 mg/L) and grown for 48 h at 28°C. An isolated colony was then inoculated on a semi-solid LB medium with the same antibiotics and grown for an additional 48 h at 28°C. Finally, the bacterial culture was suspended in 700 μL liquid LB medium containing glycerol 50% (v/v), spread on LB semi-solid medium with the appropriate antibiotics. After 24 h at 28°C, bacteria were scraped from the plate, and a fresh bacterial paste was collected at the center of a Petri dish (Fig 1), essentially as described by [26].

### 2.4. Induction of hairy roots in soybean detached leaves

In the present study, we compared the ability to generate hairy roots from detached leaves of two RKN-susceptible soybean cultivars, ‘Williams 82’ and ‘Embrapa BRS 537’, which are adapted to temperate and tropical regions, respectively. On the day of *A*. *rhizogenes* inoculation, unifoliate leaves and terminal leaflets of the trifoliate leaves were detached from 20-day-old soybean plants grown in semi-controlled greenhouse conditions, maintaining 1–2 cm of the petiole (Fig 1A). Each detached petiole was immediately inoculated with *A*. *rhizogenes* using a sterile needle (23¾ gauge) previously soaked in the freshly prepared bacterial paste (Fig 1B and 1C), as described by [26]. A small amount of bacterial paste was thus kept to cover the whole surface of the cut petiole. Two inoculated leaves were then placed per Petri dish (150 x 15 mm) containing a cotton layer covered by a wet filter paper to maintain a moist condition (Fig 1D). A glass slide was positioned below the abaxial side of the leaf to avoid direct contact with the moistened filter paper. The wounded area of the petiole was then carefully covered by sterilized wet cotton (Fig 1D), and the Petri dishes were kept under growth chamber conditions (25 ± 2°C; 12 h photoperiod; 120 μmols/m^2^/s^1^ light intensity) until the first roots exhibiting typical hairy root phenotype emerged from the petiole-wounded site.

### 2.5. Screening of eGFP‑positive soybean hairy roots

**Fig 2:**
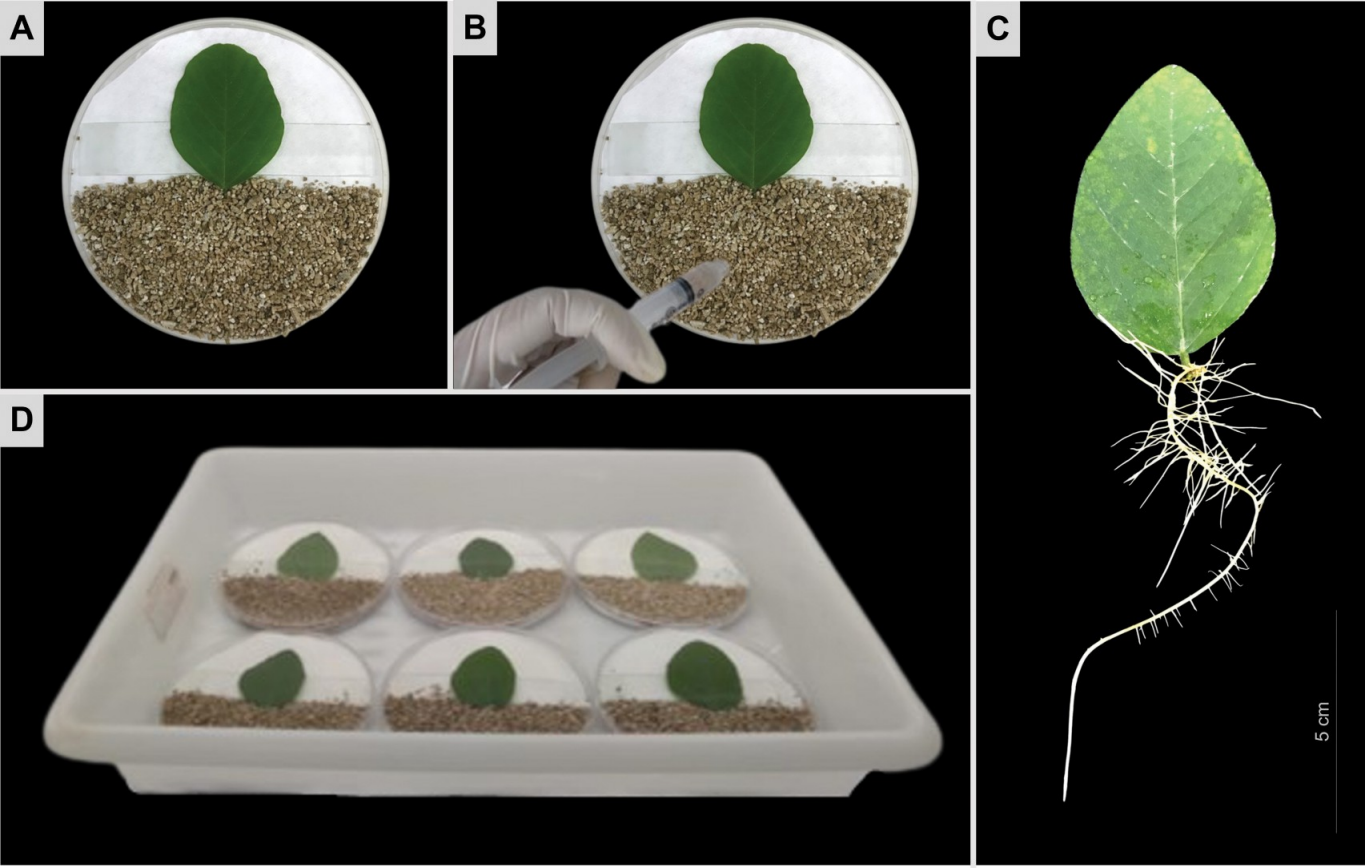
Soybean hairy root inoculation with *Meloidogyne incognita*. **(A)** Soybean (*Glycine max* cultivar ‘BRS 537’) detached leaf 30 days after *A*. *rhizogenes* transformation in Petri dish covered with vermiculite and **(B)** inoculated with a suspension of *M*. *incognita* J2 individuals when hairy roots **(C)** reached at least 10 cm in length. **(D)** Petri dishes conditioned in the tray under growth chamber conditions.

After approximately 15 days of *A*. *rhizogenes* transformation, roots exhibiting the hairy root phenotype (Fig 1E) were individually evaluated for the presence of GFP fluorescence at 488 nm under an M205 fluorescence stereomicroscope (Leica Microsystem, Wetzlar, Germany) using the GFP1 filter. Detached leaves with roots lacking fluorescence, or showing low GFP fluorescence, were considered non-transgenic and discarded, while those exhibiting GFP fluorescence (Fig 1F) were selected for further inoculation bioassays with *Meloidogyne* spp. At this point, the transformation efficiency was estimated as the number of the detached leaves that produced at least one eGFP-positive hairy root at the wounding site divided by the total number of detached leaves inoculated with *A*. *rhizogenes*. The hairy roots of the selected leaves were covered with medium-grained (5 to 8 mm) vermiculite (Fig 2A) and kept under the same growth chamber conditions.

### 2.6. Gene expression analysis in soybean hairy roots

Soybean hairy roots collected 20 to 30 days after transformation with pPZP-empty, -AdEXPA24, or -siGmPG binary vectors were frozen immediately in liquid nitrogen and stored at -80°C. Total RNA was extracted from roots using the Quick-RNA™ Plant Miniprep Kit (Zymo Research, Irvine, CA, USA), according to the manufacturer’s instructions. Next, DNA contamination was eliminated using the TURBO™ DNase Kit (Invitrogen™), followed by cDNA synthesis using the SuperScript III Reverse Transcriptase Kit and oligo(dT)_20_ primer (Invitrogen, Carlsbad, CA, USA), according to the manufacturer’s instructions.

Transgene expression in soybean hairy roots was evaluated by qRT-PCR analysis using gene-specific primer pairs (AdEXPA24 and qsiGmPG; Table 1). qRT-PCR reactions were conducted in technical triplicates for each sample on a StepOne Plus Real-Time PCR System (Applied Biosystems, Foster City, USA), as previously described by [42]. The *GmCYP2* and *GmELF1A* reference genes from *G*. *max* (Table 1) were used to normalize the expression of target genes, in accordance with [39]. The efficiency of each primer was determined by the online real-time PCR Miner tool [43], and the quantification of target genes expression was estimated by using the SATqPCR web tool [44].

### 2.7. *Meloidogyne* ssp. inoculum

*M*. *incognita* and *M*. *javanica* were multiplied on tomato (*Solanum lycopersicum* ‘Santa Clara’) plants under greenhouse conditions for three months. Eggs or freshly hatched J2s (second-stage juveniles) were extracted from roots and collected as previously described [20].

To confirm the identity of *M*. *incognita* and *M*. *javanica* inoculum, we conducted PCR analysis using specific SCAR markers developed for these RKN species [40, 41]. For that, approximately 150,000 J2 individuals were collected from diseased tomato roots, immediately frozen in liquid nitrogen into a 1.5mL microtube, and ground with a microtube pestle at room temperature. The DNA was then extracted and purified from the resulting powder by the Quick DNA Miniprep® kit according to the manufacturer’s protocol for “whole blood, serum, and plasma” (ZymoResearch, Irvine, CA, USA). PCR reactions were carried out with 5 ng of total genomic DNA in 25 μL, as described by [40], using SCAR primers (Table 1) for specific identification of *M*. *incognita* and *M*. *javanica*.

### 2.8. Hairy roots inoculation with *Meloidogyne* ssp. and infection assessment

About 30 days after *A*. *rhizogenes* transformation, when hairy roots from ‘Williams 82’ and ‘BRS 537’ soybean cultivars reached at least 10 cm in length, they were re-evaluated for the presence of GFP fluorescence as described above. GFP-positive roots were kept and inoculated with *Meloidogyne* ssp. 2,000 J2 individuals by homogeneous spreading over the region covered with vermiculite (Fig 2B and 2C), according to [26]. The Petri dishes were kept under the same growth conditions described above (Fig 2D).

Thirty days after inoculation, hairy roots were carefully removed from the vermiculite, washed in running water, weighted, and re-evaluated for GFP fluorescence, as described above. The nematode infection was assessed by counting the number of galls under a stereomicroscope (Stemi 508, Zeiss, Oberkochen, Germany). One‑way analysis of variance ANOVA followed by post‑hoc Tukey’s test (p < 0.05) were employed to examine the differences between means of root biomass, number of galls and number of galls per gram of hairy roots. The effect of each candidate on the reduction of RKN infection was statistically analyzed using Student’s t-test (p< 0.05).

## 3. Results

### 3.1. Molecular characterization of *AdEXPA24*-encoding sequence

The coding sequence of *AdEXPA24* was confirmed based on the alignment of the gene model Aradu.G235T (https://peanutbase.org/) with public available *Arachis* EXPA-encoding sequences showing high nucleotide conservation (S1A Fig). The consensus *AdEXPA24* sequence contains 759 bp encoding a polypeptide of 252 amino acids with a theoretical isoelectric point of 6.87 and molecular weight of 27.29 kDa [21]. The predicted AdEXPA24 protein showed a typical α-Expansin structure with highly conserved pattern of DPBB (positions 65 to 150) and CBM63 (positions 161 to 238) InterPro domains [45], preceded by a signal peptide in N-terminus (positions 6 to 23) (S1B Fig). The *AdEXPA24* coding sequence was successfully synthesized and cloned into the binary vector pPZP-empty [29] for further *AdEXPA24* overexpression in transgenic leaf-derived soybean hairy roots.

### 3.2. Vector construction for RNAi-mediated *GmPG* gene knockdown

In our previous comparative genomic study using 22 plant species [35], an ortholog group of 176 genes (OG0001040) encoding for Polygalacturonase (PG) was identified. Within this orthogroup, the gene representatives of three RKN-resistant legume species (*Arachis stenosperma*, *Vigna unguiculata*, and *G*. *max*) displayed a common downregulation trend upon *Meloidogyne* spp. infection. These orthologs in *A*. *stenosperma* (GDBK01014088), *V*. *unguiculata* (XM_028075749), and *G*. *max* (XM_014768222), thereby constitute promising candidates for transcript knockdown towards improved RKN-resistance legume crops. The selected 400 bp sequence (position 112 of the 5’-UTR to position 328 of the CDS region) from the *G*. *max* PG gene (*GmPG*) was cloned in sense and antisense orientations separated by a PDK intron, leading to a hairpin RNAi cassette with a calculated ΔG folding of -114.80 kcal/mol and stability confirmed *in silico* by Mfold software. The entire RNAi cassette (named siGmPG) was successfully synthesized and cloned into the binary vector pPZP-empty [29] for further *GmPG* knockdown in transgenic leaf-derived soybean hairy roots.

### 3.3. *Ex vitro* hairy root induction in soybean detached leaves

On the 15th day after *A*. *rhizogenes* transformation, the first roots started to emerge from inoculated soybean petioles, exhibiting typical hairy root phenotype characterized by fast growth, a high degree of lateral branching, and lack of geotropism [6, 46] (Fig 1E). These roots were then evaluated for GFP fluorescence (Fig 1F), and the efficiency of *A*. *rhizogenes* transformation was estimated as the percentage of soybean detached leaves that developed at least one GFP-positive hairy root.

To evaluate the best leaf explant, 15 unifoliate leaves and 15 terminal trifoliate leaflets detached from soybean ‘BRS 537’ plants were inoculated with *A*. *rhizogenes* harboring pPZP-empty. A similar transformation efficiency was obtained from both leaves (80%) and leaflets (73%), confirming that young tissues are suitable explants for hairy root induction in soybean, as previously reported [18, 47, 48]. Based on these results, unifoliate leaves were chosen as explants for the upcoming *A*. *rhizogenes* transformation assays.

Subsequent transformation assays showed that, from the 15 detached leaves of ‘Williams 82’ cultivar transformed with the pPZP-empty and 15 transformed with pPZP-AdEXPA24 vectors, 12 (80%) and 10 (66%), respectively, developed GFP-positive hairy roots. For the cultivar ‘BRS 537’, the transformation efficiencies were 80% (8/10) for detached leaves transformed with pPZP-empty vector and 70% (7/10) for pPZP-AdEXPA24. Likewise, from the 15 detached leaves transformed with the pPZP-empty and 15 transformed with pPZP-siGmPG vectors, 14 (93%) and 8 (53%), respectively, developed GFP-positive hairy roots.

These high transformation efficiencies observed for both soybean cultivars, regardless of the vector construction, confirmed that the cucumopine *A*. *rhizogenes* ‘K599’, a hypervirulent type of wild strain, is able to infect and transform a wide range of soybean cultivars [49]. Since the 90s, ‘K599’ has been successfully explored in a number of soybean *in root* biological studies, with perspectives of even wider use as its genome has been recently sequenced [50, 51].

Thirty days after *A*. *rhizogenes* inoculation, all hairy roots were GFP-positive, indicating that the earlier GFP screening was effective and ensuring that only transgenic roots would be kept and inoculated with *Meloidogyne* spp.

### 3.4. Molecular identification of *Meloidogyne* spp. inoculum

Total DNA was extracted from *M*. *incognita* and *M*. *javanica* J2 individuals collected from diseased tomato roots. Using two sets of SCAR primers previously characterized as species-specific (Table 1) [40, 41], purified DNA templates produced the expected amplification products with different patterns for the two *Meloidogyne* species tested (399 bp for *M*. *incognita* samples and 670 bp for *M*. *javanica*) (S2 Fig). These SCAR markers were selected for their specificity, easy visualization, and adequate separation by agarose gel electrophoresis of the obtained amplicons. RAPD-PCR profiling proved to be a fast and efficient approach to screen the composition of *Meloidogyne* populations prior to bioassays.

### 3.5. *Meloidogyne* spp. infection in soybean hairy roots

Thirty days after nematode inoculation, leaf-derived soybean hairy roots were re-evaluated for GFP fluorescence, and all hairy roots displayed stable GFP expression (S3A Fig). As previously reported by [52], the GFP intensity decreased as the soybean hairy roots aged, and at 60 days after *A*. *rhizogenes* transformation the fluorescence intensity was highest in actively dividing cells, such as the root meristem and nematode-induced giant cells (S3 Fig). GFP-positive roots were then collected and weighted before infection assessment. At that time, all leaves from both cultivars (‘BRS 537’ and ‘Williams 82’) displayed chlorophyll degradation and senescence, as previously observed in the screening of soybean germplasm for rust resistance using detached leaf assay [53]. Since the integrity of the detached leaves is an essential condition for the health and lifespan of the derived hairy roots under ex vitro conditions, hairy root maintenance is feasible at the most at 60 days after *Agrobacterium* inoculation. It corresponds to 30 days required for hairy root development, followed by 30 days for the nematode life cycle to complete and the infection rate to be assessed. Therefore, for optimal results, regardless of the pathogen studied, the assessment of disease symptoms in hairy roots originating from detached leaves must occur up to 30 days of the pathogen challenge.

**Fig 3:**
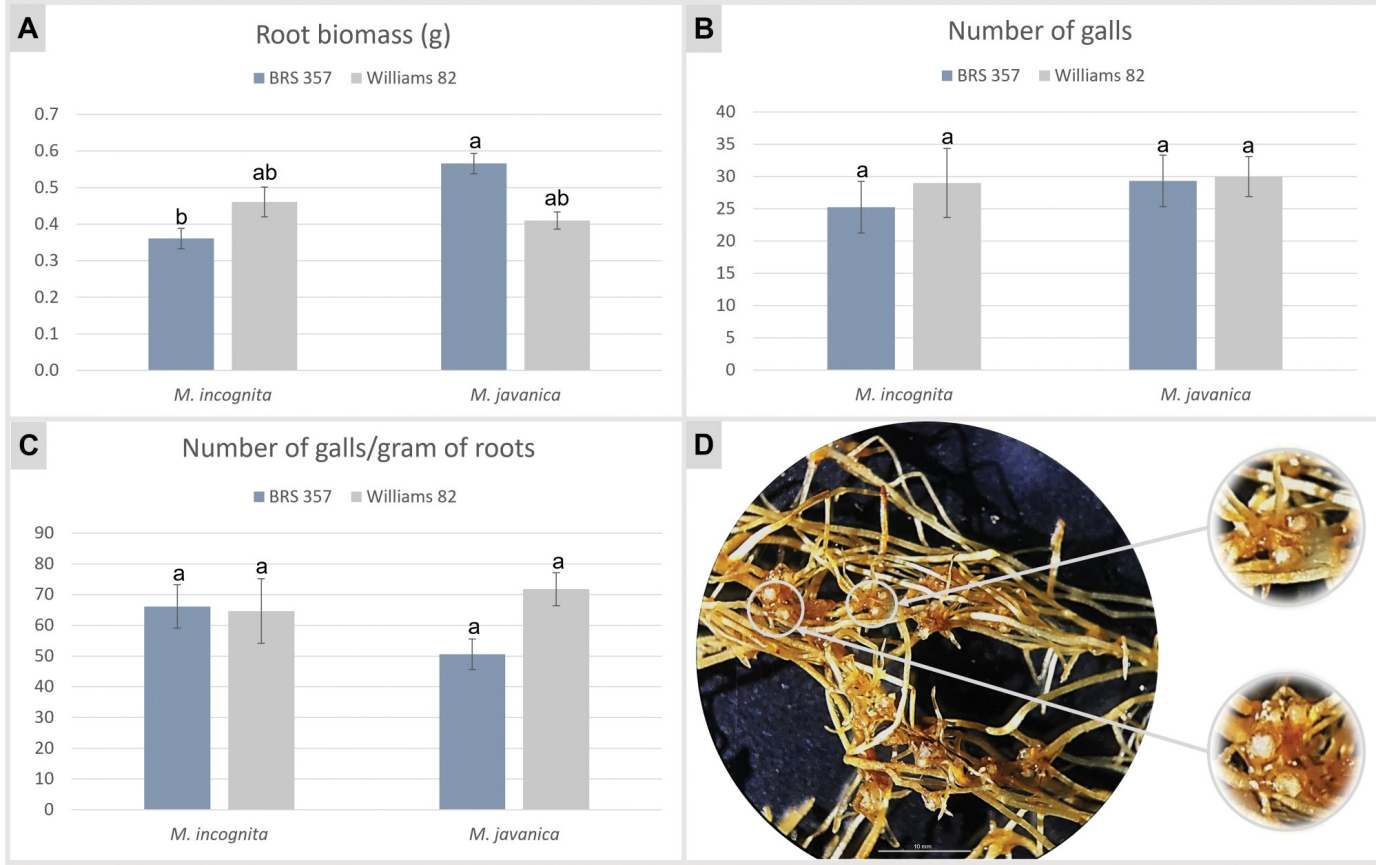
*Meloidogyne* spp. infection assessment in soybean hairy roots transformed with the pPZP-empty vector. Mean value of **(A)** fresh root biomass (in gram), **(B)** galls and **(C)** galls per gram of transgenic hairy root of two soybean cultivars (‘BRS 537’ and ‘Williams 82’), evaluated 30 days after *M*. *incognita* and *M*. *javanica* inoculation. Error bars indicate the standard deviation of samples. Different letters indicate significant differences (p < 0.05) based on ANOVA followed by Tukey’s test. **(D)** Soybean (cultivar ‘BRS 537’) hairy roots observed under a stereomicroscope, showing gall formation 30 days after *M*. *javanica* inoculation. Bars = 10 mm.

GFP-positive hairy roots from ‘BRS 537’ showed average biomass of 0.36 and 0.57 g per detached leaf transformed with pPZP-empty and inoculated with *M*. *incognita* and *M*. *javanica*, respectively (Fig 3A). These root biomass differ significantly (p ≤ 0.05), suggesting that the induction and development of hairy roots in ‘BRS 537’ cultivar were conditioned by the *Meloidogyne* species inoculated. Otherwise, ‘Williams 82’ hairy roots had average biomass that did not differ significantly, with 0.46 and 0.41 g per detached leaf when inoculated with *M*. *incognita* and *M*. *javanica*, respectively (Fig 3A).

*M*. *incognita* infection was then confirmed in soybean control hairy roots transformed with pPZP-empty by the presence of gall formation with an average of 25.23 galls and 66.16 galls per root gram in ‘BRS 537’ and 29.01 galls and 64.68 galls per root gram in ‘Williams 82’ (Fig 3B, 3C and S3 Fig). Likewise, hairy roots inoculated with *M*. *javanica* showed an average of 29.31 galls and 50.63 galls per root gram for ‘BRS 537’, and 29.99 and 71.76 in ‘Williams 82’ (Fig 3B–3D). This corroborates that the two *Meloidogyne* species can infect hairy roots derived from soybean detached leaves, regardless of the cultivar, and successfully complete their entire life cycle inside these roots.

Our findings using the control empty vector demonstrated that besides a similar *A*. *rhizogenes* transformation efficiency (average of 74%), hairy roots derived from detached leaves of both soybean cultivars could be successfully infected by *Meloidogyne* spp. and are, therefore, suitable explants for *in root* assessment of the functional role of candidate genes in soybean-RKN interaction. The susceptibility behavior of cultivated soybean to *M*. *incognita* and *M*. *javanica* is largely described in the literature, being the most common RKN species with a widespread occurrence and prevalence in tropical areas and responsible for severe damages and yield losses under field conditions [14, 54, 55].

### 3.6. *AdEXPA24* overexpression in soybean hairy roots

**Fig 4:**
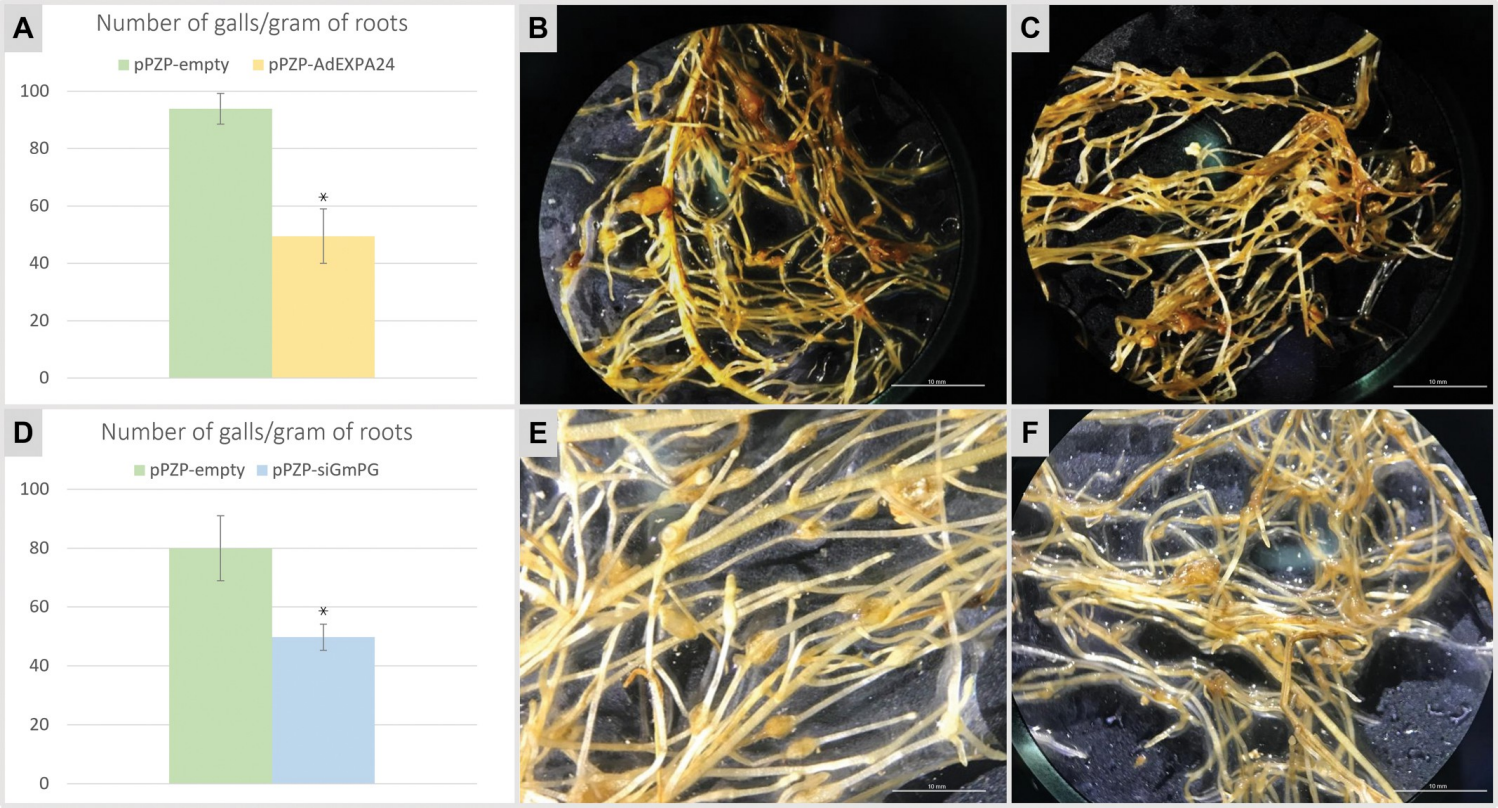
*Meloidogyne incognita* infection assessment in hairy roots overexpressing *AdEXPA24* or silencing *GmPG*. **(A, D)** Mean value of galls per gram of transgenic hairy root of soybean (cultivar ‘BRS 537’) transformed with pPZP-empty, pPZP-AdEXPA24 and pPZP-siGmPG vectors, evaluated 30 days after *M*. *incognita* inoculation. Hairy roots observed under stereomicroscope 30 days after *M*. *incognita* inoculation, transformed with *A*. *rhizogenes* harboring **(B, E)** pPZP-empty, **(C)** pPZP-AdEXPA24 and **(F)** pPZP-siGmPG vectors. Error bars indicate the standard deviation of samples. Asterisks indicate significant differences (p < 0.05; Student’s t-test) between control (pPZP-empty) and transgenic (pPZP-AdEXPA24 and pPZP-siGmPG) hairy roots.

Using the methodology established here, the candidate gene *AdEXPA24* for nematode resistance was successfully introduced and overexpressed in hairy roots of the tropical soybean cultivar ‘BRS 537’. The effect of *AdEXPA24* overexpression in RKN infection was evaluated in hairy roots at 30 days after inoculation with *M*. *incognita* analyzing the number of galls per gram of root. Then the results were compared to the control roots transformed with the pPZP-empty vector (Fig 4A). GFP-positive soybean hairy roots showed average biomass of 0.42 ± 0.19 g per detached leaf transformed with the pPZP-AdEXPA24 vector that did not differ significantly (p ≤ 0.05, t-test) from that observed in roots transformed with pPZP-empty vector (0.40 ± 0.09 g) (Fig 4B and 4C), suggesting that there is no metabolic cost of *AdEXPA24* overexpression on below-ground biomass in soybean.

The number of galls observed in hairy roots transformed with the pPZP-AdEXPA24 vector showed an average of 49.47 ± 9.47 galls per gram of roots (Fig 4A). This clearly evidences a reduction in the galls number compared to the control roots transformed with pPZP-empty (93.89 ± 5.35 galls per gram of roots) that did not express the candidate gene (Fig 4B and 4C). Therefore, the overexpression of *AdEXPA24* promoted a substantial and significant (p ≤ 0.05; t-test) reduction of 47.31%.in *M*. *incognita* infection in hairy roots derived from soybean cultivar ‘BRS 537’ (Fig 4A).

### 3.7. Downregulation of *GmPG* gene in soybean hairy roots

Likewise, the effect of *GmPG* silencing in RKN infection was evaluated in soybean hairy roots from the ‘BRS 537’ cultivar at 30 days after inoculation with *M*. *incognita* by weighting the hairy roots and counting the galls (Fig 4D). GFP-positive hairy roots showed average biomass of 0.95 ± 0.36 g per detached leaf transformed with pPZP-empty vector and 0.81 ± 0.52 with pPZP-siGmPG (Fig 4E and 4F). The biomass of roots transformed with both vectors did not differ significantly (p ≤ 0.05; t-test), indicating that the induction and development of hairy roots were not affected by the knockdown of *GmPG*. Nematode infection was further confirmed in soybean control hairy roots transformed with pPZP-empty by the presence of gall formation with an average of 79.92 ± 11.05 galls per root gram of the ‘BRS 537’ cultivar. The knockdown of *GmPG* further reduced the ability of *M*. *incognita* to infect hairy roots (49.74 ± 4.43) and led to a significant (p ≤ 0.05; t-test) reduction of 37.76% in *M*. *incognita* infection compared to the control roots transformed with pPZP-empty (Fig 4D).

### 3.8. Relative expression of *AdEXPA24* and *GmPG* in soybean hairy roots

**Fig 5:**
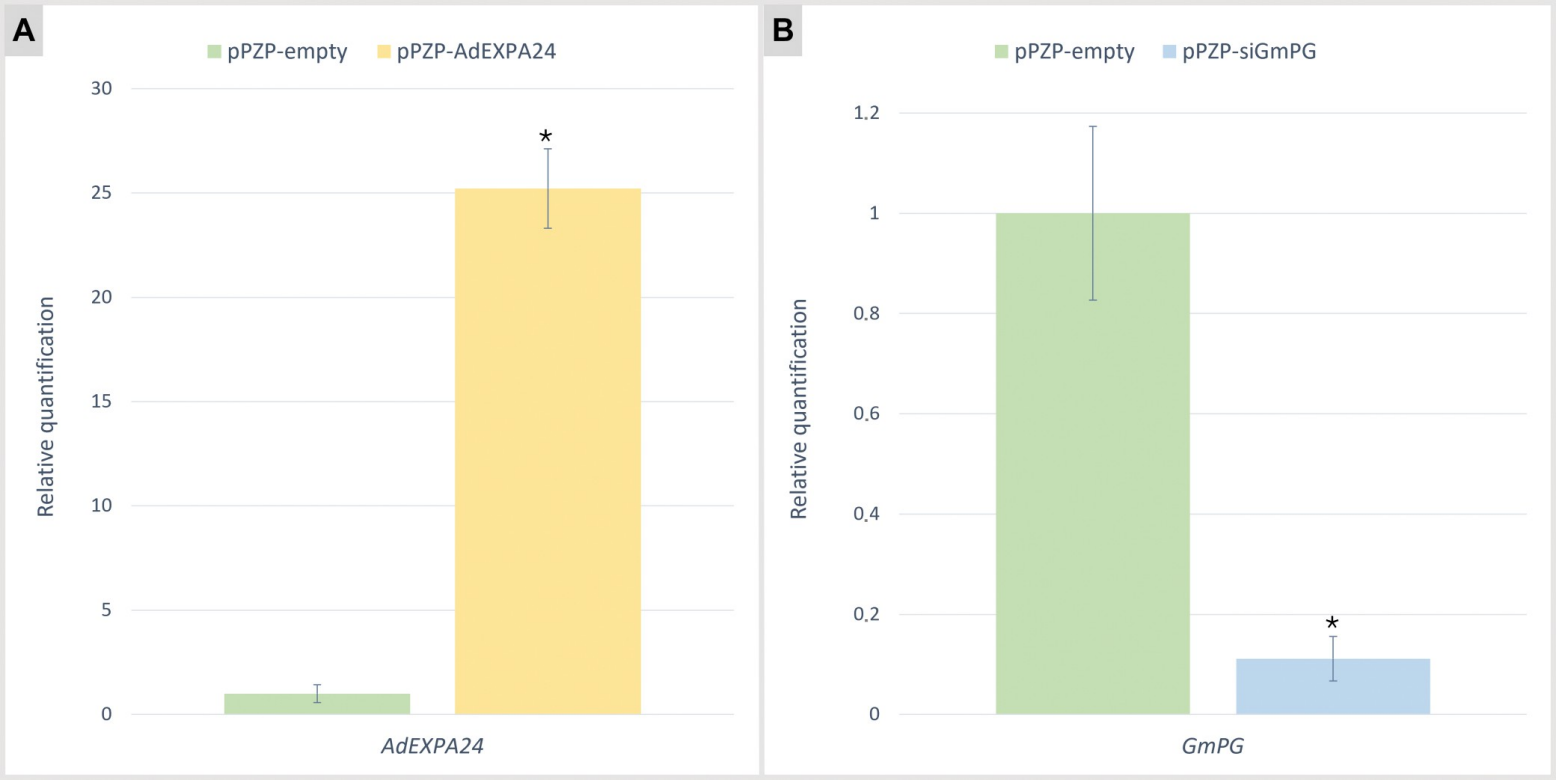
qRT-PCR analysis in transgenic soybean hairy roots. Relative quantification of *AdEXPA24* **(A)** and *GmPG* **(B)** gene expression in transgenic soybean (cultivar ‘BRS 537’) hairy roots transformed with pPZP-empty, pPZP-AdEXPA24 and pPZP-siGmPG vectors. Error bars indicate the standard deviation of three biological replicates. Asterisks indicate significant differences (p < 0.05; Student’s t-test) between control (pPZP-empty) and transgenic (pPZP-AdEXPA24 or pPZP-siGmPG) hairy roots.

The relative expression of *AdEXPA24* and *GmPG* genes was assessed by qRT-PCR analysis to confirm the transgenic status of the leaf-derived soybean hairy roots. Due to the use of a strong and constitutive AtACT2 promoter, the *AdEXPA24* transgene expression levels in pPZP-AdEXPA24 hairy roots are higher (25.2 times on average) than that observed for the soybean orthologs in the roots transformed with the control empty vector (Fig 5A). On the other hand, *GmPG* knockdown was demonstrated by an average reduction of 89% in its expression in siGmPG-transformed hairy roots compared to control roots transformed with pPZP-empty (Fig 5B). These results confirm that *AdEXPA24* is effectively overexpressed, whereas *GmPG* is almost completely silenced in transgenic soybean hairy roots.

## 4. Discussion

In soybean, detached leaves have long been the explants of choice for assessing and rapidly selecting disease resistance under growth chamber conditions, facilitating mass germplasm screening against different pathogen populations for plant breeding purposes. Therefore, detached-leaf assays have been successfully conducted to evaluate the soybean genotype’s resistance against several fungal phytopathogens (*Phakopsora pachyrhizi*; *Sclerotinia sclerotiorum; Rhizoctonia solani; Alternaria tenuissima*; *Phomopsis longicolla* and *Cercospora* spp.) and aphids (*Aphis glycines*) [53, 56–60]. Furthermore, these studies showed that the controlled environment of detached-leaf assays could overcome limitations imposed by greenhouse and field evaluations and have significant data correlation with whole plant assays. In the present study, we adapted this well-established detached-leaf method to generate hairy roots via *A*. *rhizogenes*-mediated transformation and develop a strategy for functional gene validation in soybean.

The selection of natural mutations has played a major role during the adaptation process of cultivated soybean into the tropics from its temperate origins [61], making difficult to establish a universal genetic transformation protocol that encompasses modern tropical and temperate cultivars. In addition, it is known that the efficiency of most of the standard *Agrobacterium* transformation protocols is highly genotype-dependent and restricted to certain elite soybean cultivars, constituting an important bottleneck for successful genetic transformation [62]. Therefore, for the establishment and optimization of an efficient *A*. *rhizogenes*-mediated transformation protocol of detached leaves, we worked with two RKN-susceptible conventional soybean genotypes. ‘Williams 82’, a temperate cultivar widely used as the reference soybean genome [63, 64], and ‘Embrapa BRS 537’, a highly productive tropical cultivar recently released in Brazil, whose genome has already been sequenced and added to the NCBI database [65]. Here, both soybean genotypes showed high rates (average of 74%) of *A*. *rhizogenes* transformation efficiency, summing almost ten transformed hairy roots per detached leaf. Considering that each GFP-positive hairy root has a unicellular origin and thus represents a unique transgenic event [66], an average of ten independent transformation events (cellular clones) can be obtained from a single soybean detached leaf. Our results demonstrated that the detached-leaf method is genotype-independent and generates a high number of transgenic events, which are relevant prerequisites for its adoption, and therefore is a more amenable strategy for large-scale screening of candidate genes [67].

Although a variety of soybean *A*. *rhizogenes*-mediated transformation protocols have been described, the great majority still involves *in vitro* culture steps or, most recently, the generation of whole composite plants [17, 19, 22, 25, 52, 68]. This often results in laborious, time-consuming, and costly procedures. The present detached-leaf method is an efficient and expeditious method, producing transgenic GFP-positive hairy roots just 15 days after *A*. *rhizogenes* inoculation, on average. In comparison, a standard composite plant protocol usually takes up to two months post-inoculation to produce hairy roots, whereas the most efficient methods claim to take 16 to 18 days, albeit requiring laborious steps for handling and the transplanting composite plants [17, 25]. In addition to being highly time-effective, the herein established detached-leaf system can be entirely conducted in Petri dishes, resulting in increased assay uniformity and space-saving convenience. Furthermore, since a Petri dish accommodates up to two soybean unifoliate leaves, a high number of transgenic events can be simultaneously evaluated, improving statistical accuracy and allowing massive analysis. Moreover, being conducted under controlled growth chamber conditions; this novel protocol avoids the limitations associated with the occurrence of environmental variables and pathogen co-infection, as well as space-dependency for greenhouse evaluations. Nonetheless, one should consider that each young soybean plant has only two unifoliate leaves demanding several donor plants for large-scale assays. Moreover, leaves are detached from 20-day-old soybean plants, while in other protocols younger explants, such as 7-day-old seedlings, can be used to produce transgenic roots [25]. Another constraint is that the detached leaf’s senescence process limits the lifespan of the hairy roots, and the assessment of disease symptoms in these roots must occur up to 30 days after the pathogen challenge. This short period available for phenotyping is shared by protocols using soybean whole composite plants, for which disease assessment is neither feasible for extended periods. As a result of this study, we believe that we have developed an optimized protocol encompassing a number of advantages over the current protocols for the generation of soybean hairy roots, such as high efficiency, convenience, simplicity, time-effectiveness, low cost, and space-saving.

To further validate the use of the soybean detached-leaf system for gene functional analysis, we modified the expression of two genes encoding CWMPs using opposite genetic engineering strategies: the overexpression of a heterologous *AdEXPA24*, a nematode-resistant candidate gene, and the knockdown of an endogenous *GmPG* gene potentially involved in pathogen susceptibility. Our results using the control empty vector showed that RKNs could infect and successfully complete their life cycle inside hairy roots derived from soybean detached leaves, regardless of the soybean cultivar (tropical or temperate) and the RKN species (*M*. *javanica* or *M*. *incognita*). These *Meloidogyne* species are among the most devastating and economically relevant nematodes that severely limit soybean growth and production worldwide [69]. In addition, the inoculation bioassays in roots transformed with the candidate genes for RKN control indicated that the efficiency of hairy roots induction by *A*. *rhizogenes* was not affected by either *AdEXPA24* overexpression or *GmPG* downregulation. These results support previous studies that successfully explored hairy roots for *in root* functional characterization of candidate genes involved in establishing both parasitism and symbiosis in soybean, using distinct biotechnological approaches to modify gene expression, including cis/transgene overexpression, gene knockout, gene silencing or genome editing [10, 70, 71].

We demonstrated that *AdEXPA24* overexpression significantly improved resistance in RKN-susceptible cultivars against *M*. *incognita* infection, as previously observed for soybean composite plants overexpressing other wild *Arachis* genes associated with nematode resistance [20, 21]. The functional validation of *AdEXPA24* to promote RKN resistance in soybean, a distinct heterologous background, indicates that this gene could be involved in a broad type of plant resistance, triggering non-specific defense responses against *Meloidogyne* species. The *AdEXPA24* gene, belonging to the α-Expansin subfamily, was isolated from wild *A*. *duranensis* [21, 38], and is mainly associated with abiotic stress responses, such as drought, salinity, and extreme temperatures [30]. Conversely, very few reports showed the involvement of α-Expansins in both compatible and incompatible plant-microbe interactions [35, 72–76]. Recently, some studies also demonstrated the putative role of the Expansin superfamily in mediating resistance responses to simultaneous abiotic and biotic stresses (cross-stress) as well as in defense priming processes and stress memory [35, 77–81]. Our findings support those studies by showing that *AdEXPA24* overexpression improves resistance against *Meloidogyne* sp. and suggests a similar resistance mechanism to that proposed for the overexpression of a wild *Arachis* expansin-like B gene, which leads to a general, non-specific, stress defense primed state in transgenic plants [77].

Consonantly, inoculation bioassays showed that the almost complete silencing of the *GmPG* soybean gene promoted a significant reduction in *M*. *incognita* galls formation. Plant PGs are involved in modifying pectin networks and are commonly associated with developmental processes that need cell wall disassembly, including fruit ripening, organ abscission, anther dehiscence, and pollen maturation [82]. PGs could also be implicated in host-pathogen interactions, and some studies suggested that transgenic plants deficient in PG activity exhibit increased resistance to fungal pathogens, such as *Botrytis cinerea*, *Geotrichum candidum*, and *Rhizopus stolonifera* [31, 32, 83]. In line with these findings, here we demonstrated that soybean hairy roots showed reduced levels of RKN infection due to the downregulation of an endogenous PG gene mediated by dsRNA knockdown. As suggested by [83, 84], changes in the cell wall structure and/or in DAMPs release upon pathogen invasion induced by complete to partial PG gene silencing could result in reduced susceptibility to pathogens.

The detached-leaf method established here to validate two nematode-resistant candidate genes via overexpression (gain-of-function) or dsRNA-mediated gene knockdown (loss-of-function) could also be explored for genome editing, gene knockout, promoter modification and characterization, and other applications for *in root* biology studies, in particular those concerning the establishment of both parasitism and symbiosis. In addition, this system could also be applied for the *ex vitro* propagation of nematodes, or other parasites and symbionts in soybean hairy root cultures, constituting an easy and efficient tool for functional studies on soybean-microbe interactions, overcoming limitations imposed by the need for microbe sterilization and/or maintenance of axenic *in vitro* conditions.

## 5. Conclusions

In the present work, we develop a rapid, simple, efficient, and reliable *ex vitro* methodology to generate soybean transgenic hairy roots. The detached-leaf strategy provides advantages over the current protocols for *A*. *rhizogenes*-mediated transformation of soybean by combining a number of critical conditions required for *in planta* gene characterization at the present advanced genomics era, which includes: (i) improved genetic transformation frequencies without restricted genotype dependence; (ii) high-throughput generation of transgenic events; (iii) assays conducted under space-saving and controlled environmental conditions; (iv) no-laborious steps involving tissue culture or whole plant handling; and (vi) short-time process with accurate results. This system, therefore, represents a very attractive and viable approach for functional *in root* validation of candidate genes in soybean and a promising alternative to whole composite plants or *in vitro* cultured hairy roots. Here, the application of this strategy enabled the validation of two candidate genes involved in plant cell wall disassembly to broaden the resistance of susceptible soybean cultivars against *M*. *incognita*, one of the world’s most destructive RKNs, which can be further exploited in breeding programs for nematode control.

## Supporting information

S1 Fig Molecular characterization of *AdEXPA24* gene. **(A)** Alignment of five *Arachis* spp. EXPA-encoding sequences and their consensus nucleotide sequence. **(B)** Deduced AdEXPA24 amino acid sequence and structure diagram showing DPBB and CBM63 domains and the peptide signal.
(TIFF)

S2 Fig PCR amplification patterns using species-specific primers for *Meloidogyne*. PCR amplification patterns for **(A)** *Meloidogyne incognita* and **(B)** *M*. *javanica* DNA templates using species-specific primers: SCAR-jav (lanes A1-A3, B1-B3) and SCAR-inc-K14 (lanes A4-A6, B4-B6). Lanes A1,A4: *M*. *incognita* isolates used in the present study; B1,B4: *M*. *javanica* isolates used in the present study; A5,B5: *M*. *incognita* positive controls (CTR+); A2,B2: *M*. *javanica* positive controls (CTR+); A3,A6,B3,B6: No template DNA control (negative controls; CTR-).
(TIFF)

S3 Fig Gall development in hairy roots transformed with pPZP-empty. Hairy roots **(A)** derived from soybean (*Glycine max* cultivar ‘BRS 537’) detached leaf transformed with pPZP-empty, 30 days after *Meloidogyne incognita* infection, and the developing galls imaged using **(B)** brightfield and **(C)** fluorescence stereomicroscopy. Bars = 5 mm (A) and 2 mm (B and C).
(TIFF)

S1 Raw images (PDF)

## Abbreviations

AdEXPA24: *Arachis duranensis* α-Expansin-encoding gene
CWMP: Cell Wall Modifying Protein
GmPG: *Glycine max* Polygalacturonase-encoding gene
qRT‑PCR: quantitative Reverse Transcription‑Polymerase Chain Reaction
RKN: Root-Knot Nematode
RQ: Relative Quantification
