# Supplementary figures and images for: A novel soybean hairy root system for gene functional validation
Source: PLoS One. 2023 May 18;18(5):e0285504. doi: 10.1371/journal.pone.0285504 (PMC10194865; doi:10.1371/journal.pone.0285504)

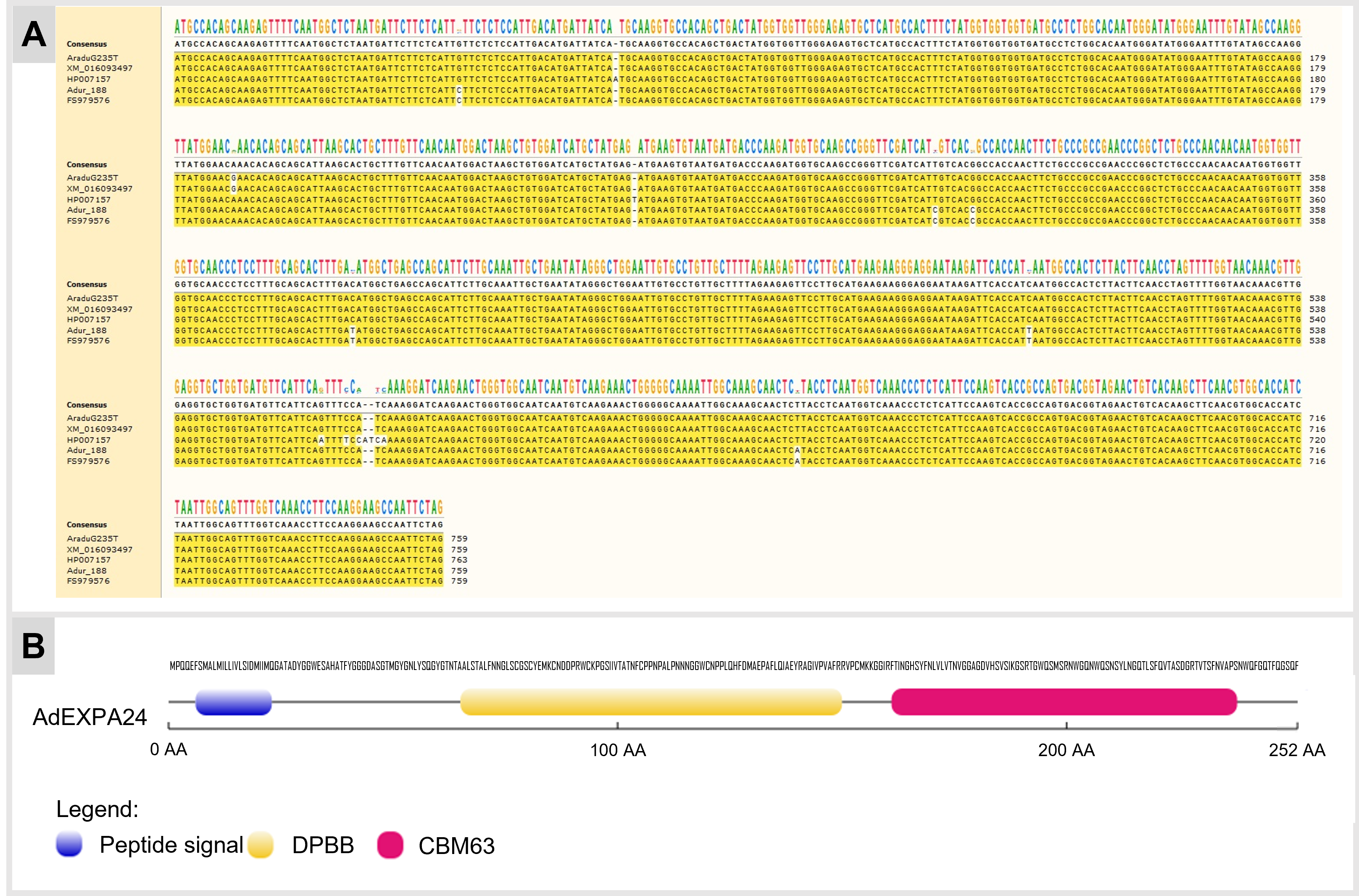

Supplement: S1 Fig — (A) Alignment of five Arachis spp. EXPA-encoding sequences and their consensus nucleotide sequence. (B) Deduced AdEXPA24 amino acid sequence and structure diagram showing DPBB and CBM63 domains and the peptide signal. (TIFF) [file pone.0285504.s001.tiff]

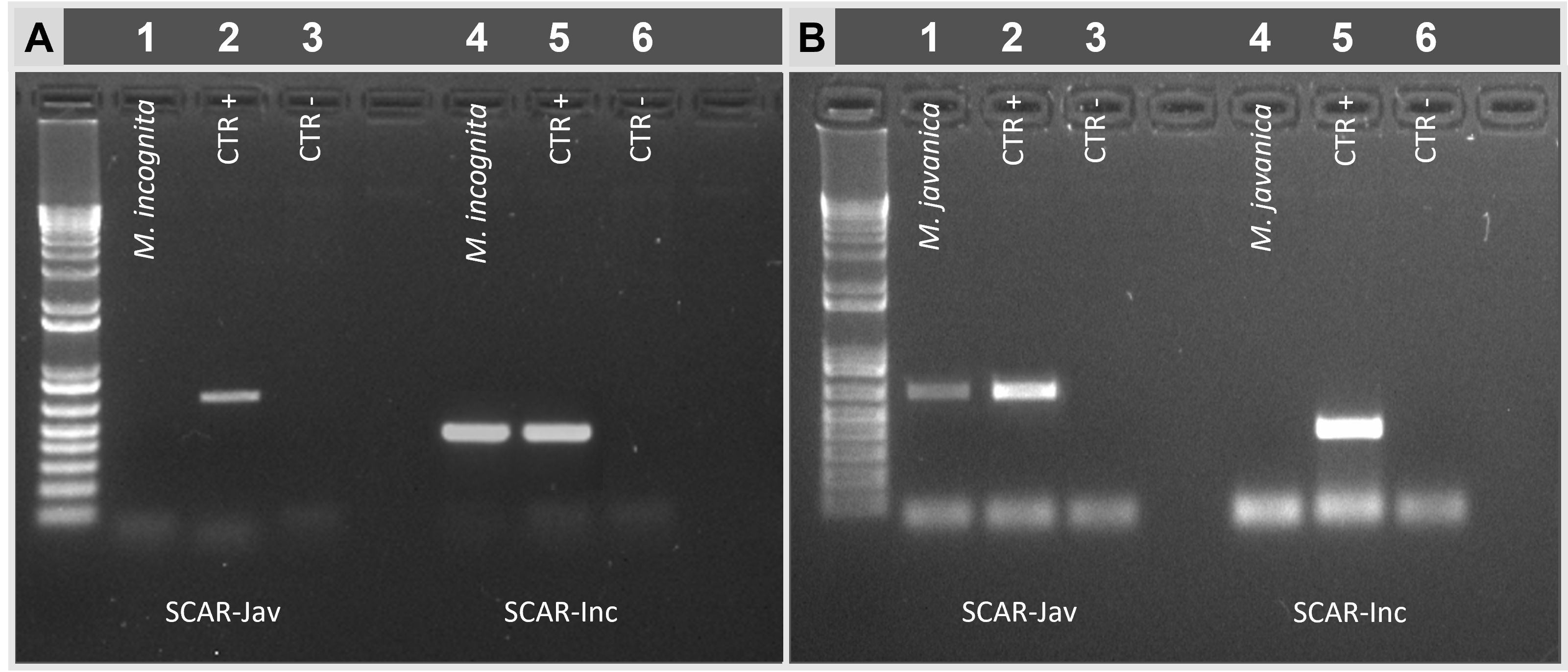

Supplement: S2 Fig — PCR amplification patterns for (A) Meloidogyne incognita and (B) M. javanica DNA templates using species-specific primers: SCAR-jav (lanes A1-A3, B1-B3) and SCAR-inc-K14 (lanes A4-A6, B4-B6). Lanes A1,A4: M. incognita isolates used in the present study; B1,B4: M. javanica isolates used in the present study; A5,B5: M. incognita positive controls (CTR+); A2,B2: M. javanica positive controls (CTR+); A3,A6,B3,B6: No template DNA control (negative controls; CTR-). (TIFF) [file pone.0285504.s002.tiff]

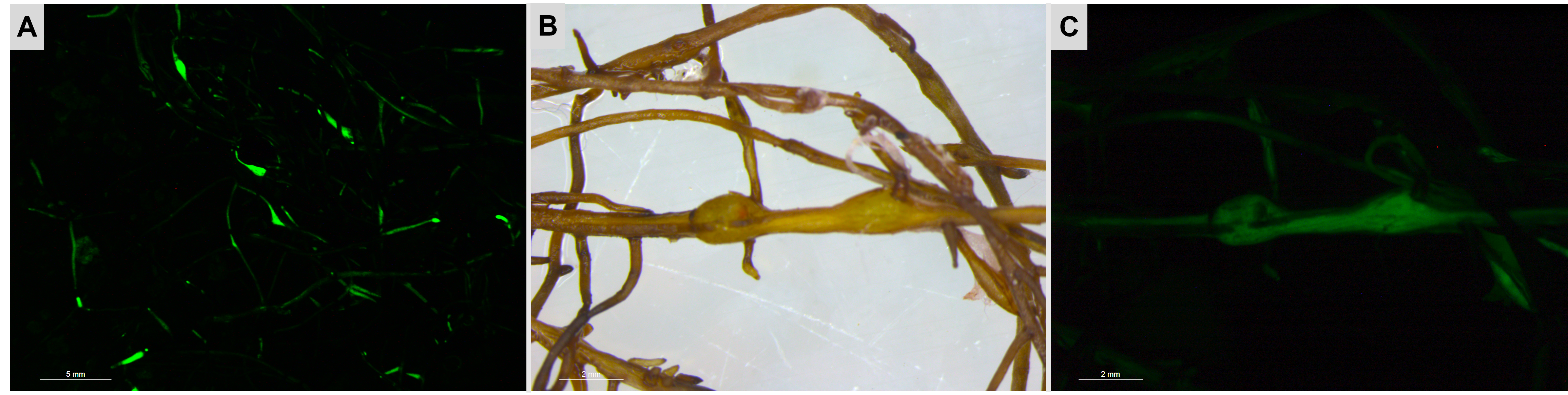

Supplement: S3 Fig — Hairy roots (A) derived from soybean (Glycine max cultivar ‘BRS 537’) detached leaf transformed with pPZP-empty, 30 days after Meloidogyne incognita infection, and the developing galls imaged using (B) brightfield and (C) fluorescence stereomicroscopy. Bars = 5 mm (A) and 2 mm (B and C). (TIFF) [file pone.0285504.s003.tiff]
