# Supplementary material for: A novel soybean hairy root system for gene functional validation
Source: PLoS One. 2023 May 18;18(5):e0285504. doi: 10.1371/journal.pone.0285504 (PMC10194865; doi:10.1371/journal.pone.0285504)
Supplement: S1 Raw images — (PDF) [file pone.0285504.s004.pdf]

Original gel image showing gel results reported in the article's supporting information Figure S2A

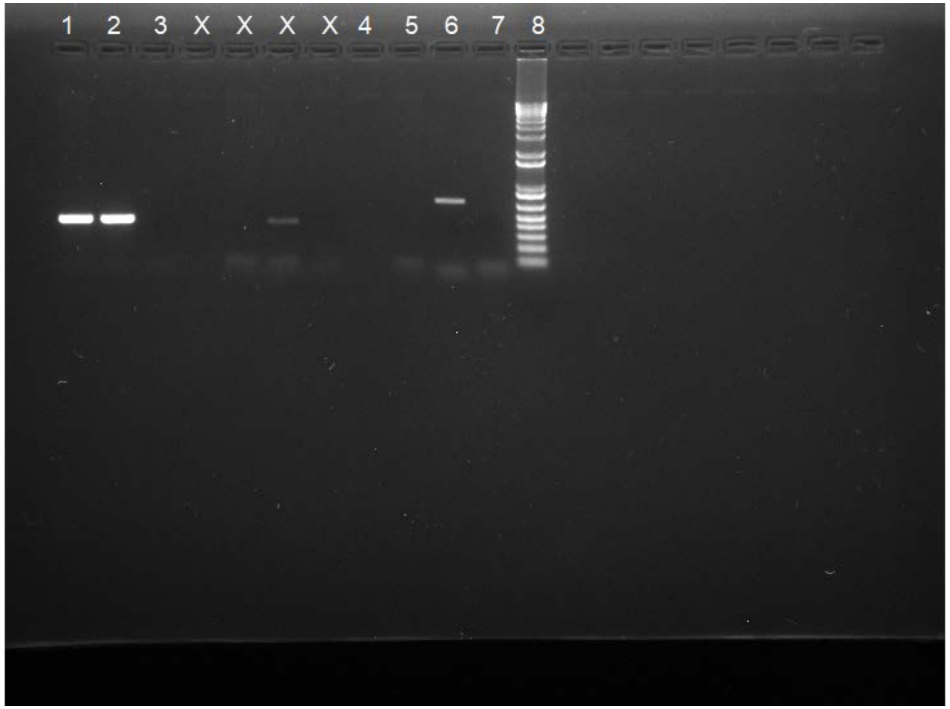

Observation: To generate the Figure S2A, the original gel image was inverted and lanes "X" were suppressed, as not related to the study.

Lane 1: *Meloidogyne incognita* (isolates used in the study) DNA template using species-specific primer SCAR-inc-K14

Lane 2: *Meloidogyne incognita* (positive control) DNA template using species-specific primer SCAR-inc-K14

Lane 3: No template DNA control (negative control) using species-specific primer SCAR-inc-K14

Lane 4: Empty lane (no sample)

Lane 5: No template DNA control (negative control) using species-specific primer SCAR-jav

Lane 6: *Meloidogyne javanica* (positive control) DNA template using species-specific primer SCAR-jav

Lane 7 = *Meloidogyne incognita* (isolates used in the study) DNA template using species-specific primer SCAR-jav

Lane 8: 1 Kb Plus DNA Ladder (Catalog # 10787026; Thermo Fisher Scientific Inc.)

- Original gel image showung gel results reported in the article's supporting information Figure S2B

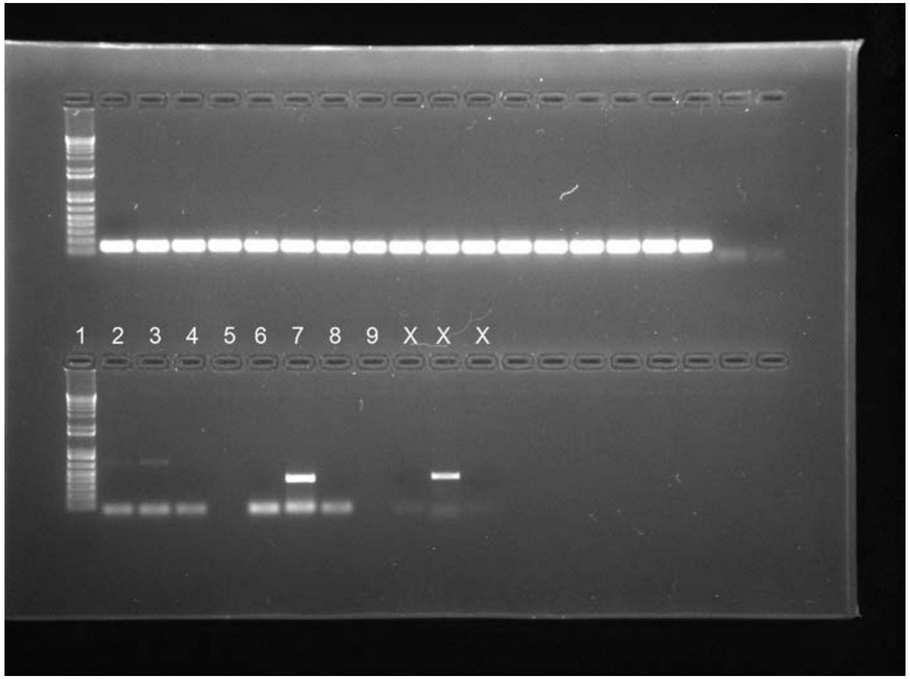

Observation: To generate the Figure S2B, the top part of the gel was eliminated and lanes "X" were suppressed, as not related to the study.

Lane 1: 1 Kb Plus DNA Ladder (Catalog # 10787026; Thermo Fisher Scientific Inc.)
Lane 2 = *Meloidogyne javanica* (isolates used in the study) DNA template using species-specific primer SCAR-jav
Lane 3: *Meloidogyne javanica* (positive control) DNA template using species-specific primer SCAR-jav
Lane 4: No template DNA control (negative control) using species-specific primer SCAR-jav
Lane 5: Empty lane (no sample)
Lane 6: *Meloidogyne javanica* (isolates used in the study) DNA template using species-specific primer SCAR-inc-K14
Lane 7: *Meloidogyne incognita* (positive control) DNA template using species-specific primer SCAR-inc-K14
Lane 8: No template DNA control (negative control) using species-specific primer SCAR-inc-K14
